# Ultrasound findings in severe COVID-19: a deeper look through the carotid arteries

**DOI:** 10.1590/0100-3984.2022.0012

**Published:** 2022

**Authors:** Camila Silva Bezerra, Alice Abath Leite, Thaís Ramos da Costa, Esdras Marques Lins, Emmanuelle Tenório Albuquerque Madruga Godoi, Lúcia Helena de Oliveira Cordeiro, Maria Cristina Falcão Raposo, Simone Cristina Soares Brandão

**Affiliations:** 1 Universidade Federal de Pernambuco (UFPE), Recife, PE, Brazil.; 2 Instituto de Medicina Integral Professor Fernando Figueira (IMIP), Recife, PE, Brazil.; 3 Faculdade Pernambucana de Saúde, Recife, PE, Brazil.; 4 Hospital Barão de Lucena, Recife, PE, Brazil.; 5 Hospital das Clínicas da Universidade Federal de Pernambuco (HC-UFPE), Recife, PE, Brazil.

**Keywords:** COVID-19, SARS-CoV-2, Ultrasonography, Carotid arteries, Carotid intima-media thickness, COVID-19, SARS-CoV-2, Ultrassonografia, Artérias carótidas, Espessura íntima-média carotídea

## Abstract

**Objective:**

To investigate vascular and perivascular abnormalities in the carotid arteries using ultrasound, as well as to evaluate their association with mortality and clinical variables in hospitalized patients with coronavirus disease 2019 (COVID-19).

**Materials and Methods:**

This was a prospective study in which 53 hospitalized patients with severe COVID-19 were evaluated and underwent carotid ultrasound. We documented the carotid ultrasound findings in these patients. Clinical, demographic, laboratory, and imaging features were analyzed and compared by statistical analysis to detect correlations between them.

**Results:**

Carotid ultrasound demonstrated luminal surface irregularity in 29 patients (55%), carotid plaques in 30 (57%), perivascular infiltration in four (8%), and increased intima–media thickness (IMT) in 31 (58%). Of the 31 patients with increased IMT, 19 (61%) died, and the association between increased IMT and COVID-19–related mortality was significant (*p* = 0.03). Logistic regression showed that the risk of death was 85% in patients who had increased IMT in combination with acute kidney injury at admission or a history of chronic kidney disease (*p* < 0.05).

**Conclusion:**

In hospitalized patients with severe COVID-19, carotid ultrasound can show increased IMT, luminal surface irregularity, carotid plaques, and perivascular infiltrates. The combination of increased IMT and kidney damage appears to increase the risk of death in such patients.

## Abstract (pt)

**Objetivo:**

Investigar anormalidades vasculares e perivasculares nas artérias carótidas por meio de ultrassonografia e avaliar sua associação com mortalidade e variáveis clínicas em pacientes hospitalizados com COVID-19.

**Materiais e Métodos:**

Neste estudo prospectivo, 53 pacientes hospitalizados com COVID-19 grave foram avaliados e submetidos a ultrassonografia de carótida. Descrevemos os achados ultrassonográficos de carótida nesses pacientes. As correlações de características clínicas, demográficas, laboratoriais e de imagem foram analisadas e comparadas por meio de análise estatística.

**Resultados:**

A ultrassonografia carotídea demonstrou irregularidade da superfície luminal em 29 pacientes (55%), placas carotídeas em 30 pacientes (57%), infiltração perivascular em quatro pacientes (7,5%) e aumento da espessura médio-intimal (EMI) em 31 pacientes (58%). Dos pacientes com EMI aumentada, 19 (61%) morreram, com associação observada entre EMI aumentada e mortalidade por COVID-19 (*p* = 0,03). Um modelo de regressão logística mostrou que a probabilidade de óbito foi de 85% em pacientes com EMI aumentada e história de nefropatia crônica ou lesão renal aguda na internação (*p* < 0,05).

**Conclusão:**

Aumento da EMI, irregularidade da superfície luminal, placas carotídeas e infiltrados perivasculares foram encontrados na ultrassonografia carotídea em pacientes hospitalizados com COVID-19 grave. O aumento da EMI associado a danos nos rins pode aumentar o risco de morte.

## INTRODUCTION

As of February 4, 2022, coronavirus disease 2019 (COVID-19) had caused approximately 386 million infections and 5.7 million deaths^(1)^. Severe acute respiratory syndrome coronavirus 2 (SARS-CoV-2) proved to be a complex infectious agent that is responsible for extrapulmonary presentations, including renal, cutaneous, gastrointestinal, neurological, and cardiovascular changes^(2,3,4,5,6,7)^

*In vitro* experiments and autopsy studies have shown that SARS-CoV-2 binds to host angiotensin-converting enzyme 2 (ACE2) receptors^(8,9)^. Therefore, tissues with high ACE2 surface expression are considered susceptible to direct viral infection^(10)^. It is known that ACE2 is widely distributed throughout the body and abundantly expressed on the surface of the vascular endothelium^(5,9,10)^.

Postmortem examinations of patients with severe COVID-19 have revealed endotheliitis and other vascular changes, such as pulmonary intussusceptive angiogenesis, cell necrosis, and microthrombi^(11,12)^. Alterations in endothelial glycocalyx thickness have also been reported^(13)^. Those findings support the concept that COVID-19 is a vascular disease, the endothelium playing a central role in its pathophysiology^(11,13,14,15)^.

Carotid ultrasound is a widely available, noninvasive tool for the evaluation of vascular abnormalities, including changes in intima–media thickness (IMT) and other pathologic changes in the vessel walls^(16)^. Despite the advantages of this imaging method, there have been, to our knowledge, no ultrasound studies on carotid manifestations related to SARS-CoV-2 infection, except for a few case reports. The aim of this study was to identify and characterize IMT changes and vascular abnormalities in patients with severe COVID-19 through carotid ultrasound and to correlate those abnormalities with clinical variables and with mortality.

## MATERIALS AND METHODS

This was a prospective, cross-sectional, multicenter study conducted at three hospitals that are referral centers for the care of patients with COVID-19 in Brazil. We evaluated a convenience sample of inpatients ≥ 18 years of age—including intensive care unit (ICU) patients—all of whom tested positive for SARS-CoV-2, by reverse-transcriptase polymerase chain reaction, at one of the participating hospitals between July 2020 and February 2021.

The study was approved by the Research Ethics Committee of the Federal University of Pernambuco Hospital das Clínicas (Reference no. 34736620.6.0000.8807). All participating patients or their surrogates gave written informed consent.

### Image acquisition and ultrasound protocol

Three board-certified radiologists (with 4–10 years of experience) performed ultrasound examinations at the bedside, using LOGIQ systems (GE Healthcare, Waukesha, WI, USA) equipped with high-frequency (12-mHz) linear transducers (12L-RS; GE Healthcare), each patient being examined by at least two radiologists together on site. The radiologists were aware of the SARS-CoV-2-positive status of the patients but were blinded to other clinical and laboratory data.

The carotid ultrasound protocol included assessment of the common carotid artery, carotid bifurcation, and at least 2 cm of the internal and external carotid arteries. The vessels were evaluated using grayscale and color Doppler studies to identify the following: increased IMT, luminal surface irregularity, plaques, plaque characteristics, and thrombi.

### Data collection and image reading

The researchers who collected the ultrasound data stored those data on local electronic devices and subsequently evaluated them in a clinical-radiological environment with the other members of the research team. The images were reviewed by all three radiologists, and findings were included in the study only when commonly observed by at least two of the three. All discrepancies regarding the initial interpretation of the data were resolved by consensus through consultation with a vascular ultrasound specialist, a vascular surgeon, and a cardiologist. Because some aspects of an ultrasound study can be subjective and because there was considerable variation in age among the patients evaluated (most being < 65 years of age), specific criteria were used in the ultrasound examinations and in the image review, with the objective of reducing the number of false-positive results. We measured IMT, defined as the distance between two hyperechoic lines corresponding to the lumen–intima and media–adventitia interfaces^(16,17)^, in a plaque-free segment of the posterior wall of the common carotid, and IMT was considered to be increased if the mean value between three manual measurements was greater than the 75th percentile reported for the general population of Brazil^(16,17,18)^. In addition, we evaluated irregularity of the luminal surface of the carotid arteries in grayscale, comparing the findings in the longitudinal and axial axes to avoid possible ultrasound artifacts. Furthermore, we defined an atheromatous plaque as a focal structure extending at least 0.5 mm into the vessel lumen, measuring ≥ 50% of the value of the adjacent IMT measurement, an IMT measurement of > 1.5 mm, or any combination of those^(16,19)^. Moreover, the carotid plaques were divided into three subgroups and characterized, on the basis of their echogenicity, as follows: hypoechoic (echogenicity similar to that of blood); isoechoic (echogenicity similar to that of the sternocleidomastoid muscle); or hyperechoic (“whiter” than the adjacent muscle). In that last subgroup, we also included hyperechoic plaques producing posterior acoustic shadows (corresponding to calcifications).

After the imaging data had been reviewed, clinical, demographic, and laboratory data were collected from electronic and physical records by researchers who were blinded to the imaging findings, and those data were subsequently reviewed by the three radiologists. The following comorbidities were evaluated: hypertension; diabetes mellitus; acute kidney injury at admission or a history of chronic kidney disease; coronary artery disease; and history of stroke or deep vein thrombosis.

### Statistical analysis

Demographic, clinical, laboratory, and imaging data were compared by using statistical tests such as Pearson’s chi-square test and the Mann-Whitney test. To explain the outcome, logistic regression models were used in order to evaluate imaging variables (IMT, plaques, and luminal surface irregularity) together with the clinical data (age, sex, ICU admission, and comorbidities), and pooled analyses were adjusted. Values of *p* < 0.05 were considered indicative of a significant difference. All statistical analyses were performed with the IBM SPSS Statistics software package, version 20.0 (IBM Corp., Armonk, NY, USA).

## RESULTS

A total of 210 patients who tested positive for infection with SARS-CoV-2 were admitted to the sectors evaluated in the three participating hospitals, and 166 (79%) of those patients were admitted to the ICU. Among the 210 patients evaluated, the mean age was 60 ± 17 years (range, 19–99 years) and 122 (58%) were men.

A total of 53 patients (31 men; 22 women) underwent carotid ultrasound, and 43 (81%) were admitted to the ICU. The mean age of those patients was 60 ± 15 years (range, 24–89 years). The mean time from admission to carotid ultrasound was 8.4 ± 8.0 days, and nine patients underwent follow-up ultrasound. The characteristics of the patients who underwent carotid ultrasound are listed in Table 1.

**Table 1:** Demographic and clinical characteristics of hospitalized patients with severe COVID-19.

Characteristic	ICU (n = 43)	Non-ICU (n = 10)	P
Age, mean ± SD	59 ± 15	61 ± 15	0.90*
Sex, n (%)			
Male	25 (82)	6 (18)	> 0.99^†^
Female	18 (81)	4 (19)	
Days from admission to outcome, mean ± SD Comorbidities, n (%)	22.8 ± 15.1	27.7 ± 22.4	0.65*
Hypertension	28 (65)	6 (60)	> 0.99^†^
Diabetes	26 (60)	5 (50)	0.71^†^
Asthma/COPD	9 (21)	2 (20)	> 0.99^†^
Coronary disease	4 (9)	1 (10)	> 0.99^†^
History of malignancy	9 (21)	2 (20)	> 0.99^†^
Autoimmune disease	1 (2)	0 (0)	> 0.99^†^
Kidney disease	22 (51)	2 (20)	0.09^†^
HIV infection	1 (2)	2 (20)	0.08^†^
Thrombotic complications, n (%)			
History of stroke	4 (9)	0 (0)	> 0.99^†^
In-hospital stroke	2 (5)	0 (0)	> 0.99^†^
History of deep vein thrombosis	17 (50)	2 (20)	0.41^†^
Invasive ventilation, n (%)	33 (77)	2 (22)	0.003^†^
Outcome, n (%)			
Discharge	19 (44)	8 (80)	0.07^†^
Death	24 (56)	2 (20)	

ICU, intensive care unit; COPD, chronic obstructive pulmonary disease.

* Mann-Whitney test; ^†^ Fisher’s exact test.

The most common findings were increased IMT (in 58%), diffuse luminal surface irregularity (in 55%), and plaques (in 57%). The mortality rate was significantly higher among the patients with increased IMT than among those without (61% vs. 32%; *p* = 0.03). Among the patients with increased IMT, ages ranged from 48 to 85 years and the mean IMT was 0.82 mm (range, 0.5–2.1 mm).

**Figure 1:**
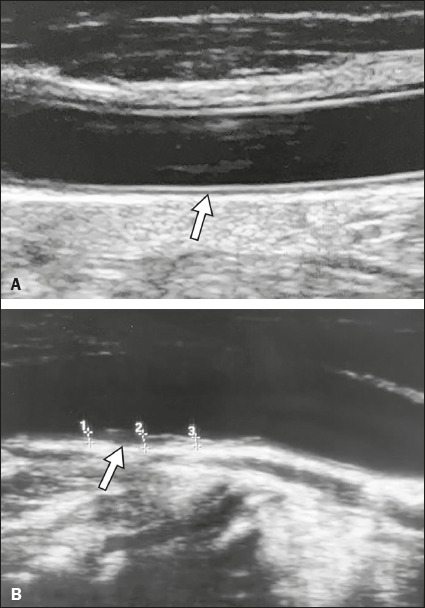
**A**: Grayscale carotid ultrasound image of a 52-year-old, HIV-infected man with COVID-19, showing no evidence of ultrasound IMT abnormalities (arrow). **B**: Follow-up carotid ultrasound study performed 12 days after the first examination, showing increased IMT and luminal surface irregularity (arrow).

One patient in whom the initial ultrasound findings were normal (IMT = 0.5 mm) underwent follow-up ultrasound due to clinical worsening. The follow-up ultrasound showed increased IMT (1.2 mm) and luminal surface irregularity (Figure 1). The patient was transferred to the ICU and progressed to death.

**Figure 2:**
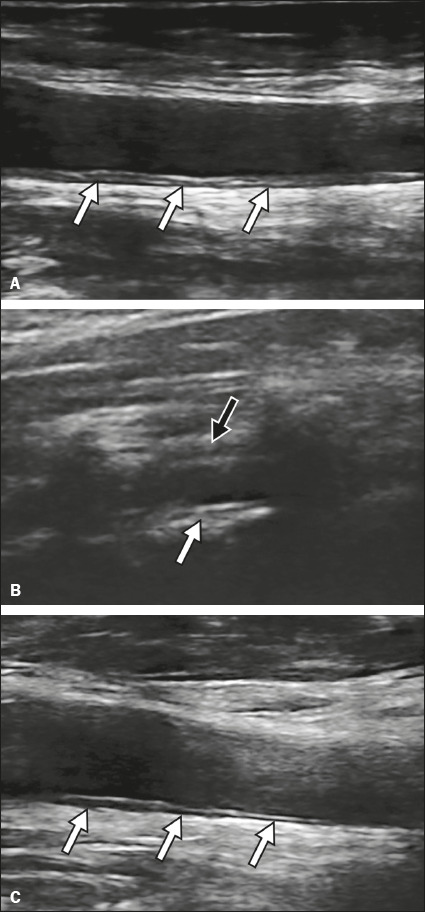
A 50-year-old man with COVID-19. Grayscale ultrasound images showing a thickened, irregular common carotid artery (white arrows) on the longitudinal axis (**A**) on day 3 after the onset of symptoms. In **B**, the vertebral artery is also diffusely thickened (white arrow), with signs of perivascular infi ltration (black arrow). In **C**, there was slightly less perivascular infl ammation (arrows) on day 10, coinciding with clinical improvement after treatment.

Another patient admitted to the ICU underwent carotid ultrasound on postadmission day 3 and was found to have perivascular edema and increased IMT (1.03 mm). A follow-up ultrasound examination performed on postadmission day 10 (after treatment) showed decreases in the perivascular edema and in IMT, which coincided with an improved clinical condition (Figure 2).

**Figure 3:**
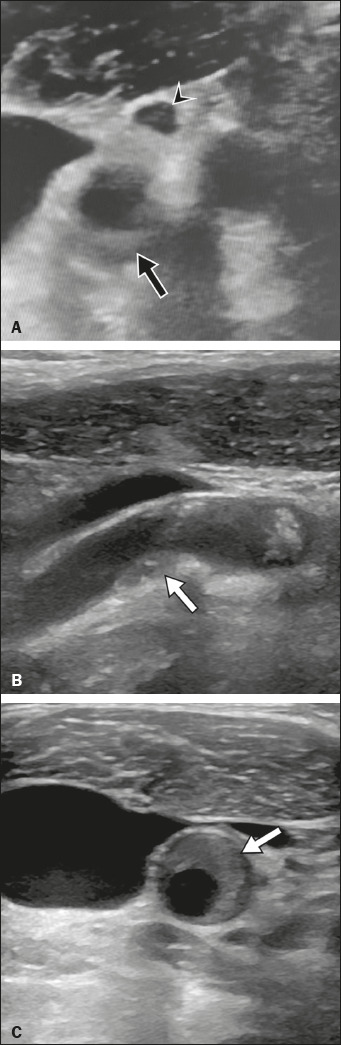
**A:** Grayscale carotid ultrasound image of a 67-year-old man with COVID-19, showing perivascular infi ltrate near the carotid bifurcation, more prominent in planes surrounding the internal carotid artery (arrow). Note also the small lymph node with reactive characteristics (arrowhead). **B**,**C**: Plaques of different sizes and echogenicity (arrows), predominantly hypoechoic or isoechoic, extending from the internal carotid and carotid bifurcation to the common carotid, which presents a diffuse, irregular increase in IMT (arrows). These characteristics are usually seen in TIPIC syndrome.

Four patients showed increased IMT accompanied by signs of perivascular infiltration, which created a “hazy” aspect in the fat surrounding the vessel. One of those four patients had eccentric perivascular infi ltration near the carotid bifurcation, together with a diffuse increase in IMT and luminal narrowing, as well as isoechoic and hypoechoic plaques, an aspect usually seen in transient perivascular infl ammation of the carotid artery (TIPIC) syndrome (Figure 3).

**Figure 4:**
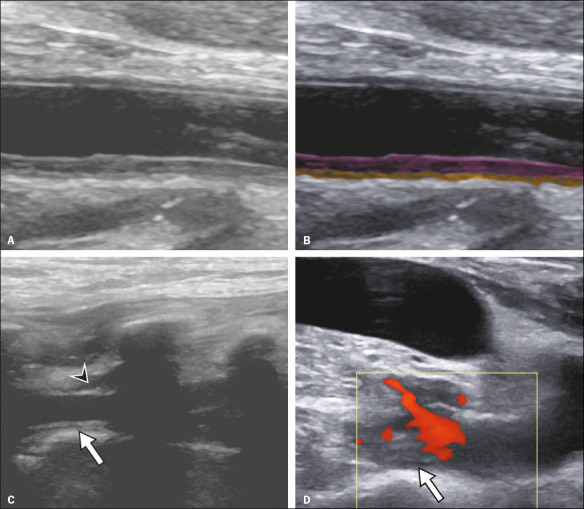
A 50-year-old woman with COVID-19. Grayscale ultrasound image (**A**) and its respective annotated image (**B**). In **B**, note the common carotid with a diffuse increase in IMT highlighted in pink and perivascular infiltration highlighted in orange. In **C**, the vertebral artery is also thickened (arrow), with signs of perivascular infiltration (arrowhead). In **D**, color Doppler study showing a thrombus in the subclavian artery.

An additional study of the vertebral arteries was performed in three patients. All three of those patients were found to have diffuse wall thickening in those vessels and in the carotid arteries, as well as signs of perivascular inflammation (Figure 4).

**Figure 5:**
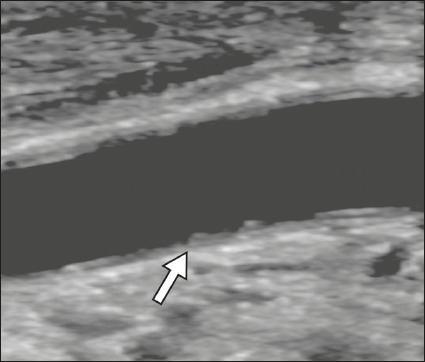
Carotid ultrasound of a 59-year-old woman with COVID-19, showing marked, diffuse luminal surface irregularity (arrow).

Carotid ultrasound revealed diffuse luminal surface irregularity in 29 (55%) of the 53 patients (Figure 5). Of those 29 patients, 13 (45%) progressed to death.

Carotid plaques were observed in 30 (57%) of the 53 patients evaluated. Of those 30 patients, nine (30%) had hypoechoic plaques in the right carotid; eight (27%) had hypoechoic plaques in the left carotid; three (10%) had isoechoic plaques in the right carotid; and one (3%) had isoechoic plaques in the left carotid. Hyperechoic/calcified plaques were observed in the right carotid in ten patients (33%) and in the left carotid in 16 (53%). It should be borne in mind that more than one type of plaque can be present in the same patient.

Vascular coagulation-related abnormalities were also identified, including thrombosis of the subclavian artery in one patient (Figure 4), thrombus in the right brachiocephalic vein in one, thrombus in the jugular vein in one, and thrombus in a central venous catheter in two.

Among the 53 patients who underwent carotid ultrasound, mechanical ventilation was required in 35 (66%), and 21 (60%) of those 35 patients were men. Hemodialysis was required in 22 patients (42%), of whom 14 (64%) were men. Of the 53 patients evaluated, 26 (49%) died, and 16 (61%) of those deaths were in men. In our sample, the mortality rate was higher among the men than among the women (52% vs. 45%). The main results and their associations with the outcomes are shown in Table 2.

**Table 2:** Variables and their association with the outcome among hospitalized patients with severe COVID-19

Variable	Outcome		Total	P
	Discharge	Death		
Sex				
Male, n (%)	12 (55)	10 (45)	22 (100)	0.19*
Female, n (%)	15 (48)	16 (52)	31 (100)	
Age, mean ± SD	57 ± 17	63 ± 11	60 ± 15	0.33^†^
Type of hospitalization				
ICU, n (%)	19 (44)	24 (56)	43 (100)	0.04*
Non-ICU, n (%)	8 (80)	2 (20)	10 (100)	
Hospital stay (days), mean ± SD	24.4 (20.4)	22.8 (11.4)	23.6 (16.3)	0.33^†^
IMT				
Yes, n (%)	12 (39)	19 (61)	31 (100)	0.03*
No, n (%)	15 (68)	7 (32)	22 (100)	
Luminal surface irregularity				
Yes, n (%)	16 (55)	13 (45)	29 (100)	0.73*
No, n (%)	9 (50)	9 (50)	18 (100)	
Plaque(s)				
Yes, n (%)	16 (53)	14 (47)	30 (100)	0.69*
No, n (%)	11 (48)	12 (52)	23 (100)	

ICU, intensive care unit.

* Pearson’s chi-square test. ^†^ Mann-Whitney test.

The risk of death was estimated to be greater in patients with increased IMT (odds ratio: 4.1; 95% CI: 1.1– 15.8; *p* = 0.04), as well as in those with acute kidney injury at admission or a history of chronic kidney disease (odds ratio: 8.9; 95% CI: 2.4–33.7; *p* = 0.001). Patients with both had an 85% risk of progressing to death. For the patients with severe COVID-19 who had none of those risk factors, the risk of death was estimated to be 13%.

## DISCUSSION

In the present study, the most common carotid ultrasound finding in patients with severe COVID-19 was increased IMT. Our results suggest that increased IMT is associated with an increased risk of death in such patients and worsens the prognosis in those with concomitant kidney disease. We also observed an increase in IMT after clinical worsening in a patient with normal findings on a previous carotid ultrasound, as well as demonstrating decreases in pathologic alterations (perivascular edema and IMT) on the follow-up carotid ultrasound of another patient, which coincided with improvement in the clinical condition after treatment.

Although the explanations for our findings are not yet well defined, some hypotheses can be considered, including direct viral infection, because ACE2 expression is abundant in the vascular endothelium, autopsy studies having indicated that SARS-CoV-2 has a direct inflammatory effect^(8)^, as well as causing endothelial damage associated with the presence of intracellular viral particles and the occurrence of cell membrane ruptures^(12)^. Viremia in the endothelium could contribute to worsening the prognosis in patients with kidney disease and increased IMT, as observed in the present study, together with dysregulation of the renin–angiotensin–aldosterone system and immune response^(20)^.

Increased IMT is also observed in inflammatory processes, such as TIPIC syndrome^(21,22)^, vasculitis, and other infectious processes, including infection with HIV and with coronaviruses other than SARS-CoV-2^(17,23)^. The hyperinflammatory state related to the “cytokine storm” in patients with severe COVID-19 might be linked to increased IMT. That inflammatory mechanism might justify the increased IMT in the patient who previously had no such alteration. It might also be associated with clinical and radiological improvement in the other patient with reduced perivascular inflammatory process after therapeutic management. Signs of perivascular infiltration have been reported in vasculitis and in TIPIC syndrome, and it has been suggested that these findings are associated with inflammation or with the expression of an autoimmune process^(21)^.

Luminal surface irregularity and plaques were also observed in our sample. Luminal surface irregularity on ultrasound may be associated with atherogenic factors or with endotheliitis caused by the SARS-CoV-2 infection. Hypoechoic plaques are usually found in vasculitis, especially when the vasculitis is accompanied by perivascular infiltration and a diffuse increase of IMT^(21,22,24,25)^. Such hypoechoic plaques might also be related to activation of endothelial function by SARS-CoV-2 infection, in the sense of promoting atherosclerosis, as well as the participation of the inflammatory process and the contribution of hematopoiesis after exposure to stressor stimuli, the combination of which favors atherogenesis^(26)^.

Traditional cardiovascular risk factors are also associated with increased IMT and other pathologic carotid wall findings^(16)^, which gives rise to the following questions: Is the IMT increase in patients with COVID-19 a preexisting, transitory, or permanent finding?; and Does that thickening represent an additional cardiovascular risk for such patients? Because our study was conducted between the first and second waves of the COVID-19 pandemic, a period of increased in-hospital morbidity and mortality in Brazil^(27)^, few patients were in condition to undergo follow- up ultrasound. Therefore, further studies are needed in order to clarify our findings.

Imaging features related to thrombosis support the idea that hospitalized patients with COVID-19 are often in a state of hypercoagulation^(28)^. Previous studies involving histological analysis of pulmonary vessels in patients with COVID-19 have reported findings such as diffuse thrombosis, microangiopathy, occlusion of small pulmonary vessels, and microthrombi in alveolar capillaries^(11,12,29,30)^.

As for clinical practice, our findings suggest that evaluation of the carotid arteries may facilitate the management of severe COVID-19 and help identify possible complications. Increased IMT might be a predictor of a worse outcome of the disease, especially in patients with a history of chronic kidney disease or acute kidney injury. It is also possible that carotid ultrasound will improve the assessment of treatment response, through patient screening and follow-up.

Our study has some limitations. The most important are the small sample size and the fact that the study design did not allow the establishment of a causal relationship. It should be borne in mind that performing ultrasound in critically ill patients with COVID-19 requires time for donning and removing personal protective equipment, as well as the use of strict protocols to minimize the exposure of the team. It was also difficult to obtain adequate ultrasound access, due to the presence of cervical sutures and monitoring devices, as well as clinical instability and prone positioning. In addition, some data were missing from the medical records, which limited the clinical-radiological correlation. Furthermore, our study lacked a control group, because the pandemic forced health care facilities to adapt to changing needs, resulting in a sudden drop in the number of non-COVID-19-related hospitalizations during the lockdown. Our study was conducted at hospitals that are referral centers for the care of patients with COVID-19, where there were even fewer non-COVID-19-related admissions. Despite these limitations, it was possible to document and evaluate the carotid ultrasound findings in critically ill patients with COVID-19.

In conclusion, we observed vascular and perivascular changes on carotid ultrasound in patients with severe COVID-19, the main findings in those patients being increased IMT, luminal surface irregularity, perivascular infiltrates, and carotid plaques. The combination of increased IMT and kidney damage appears to increase the risk of death in such patients. Subsequent studies evaluating the endothelium in the different phases of COVID-19, including patients with the mildly symptomatic and asymptomatic forms of the disease, could provide more information and are therefore warranted.
